# CORRELATES OF RETIREMENT EXPECTATIONS AMONG BABY BOOMERS IN US AND SOUTH KOREA: APPLICATIONS OF TREE-BASED METHODS

**DOI:** 10.1093/geroni/igad104.0879

**Published:** 2023-12-21

**Authors:** Linh Dang, Briana Mezuk

**Affiliations:** University of Michigan, Ann Arbor, Michigan, United States; University of Michigan, Ann Arbor, Michigan, United States

## Abstract

75 million US Baby Boomers are expected to retire by 2030. Retirement decisions are a result of a complex interplay of multiple psychosocial factors, including individual (demographics, health characteristics), family (marital status, spousal characteristics), work-related factors (job characteristics, workplace environment), and cultural contexts surrounding retirement. However, traditional regressions of retirement expectations limit the inclusion of a large set of factors and their interactions. This study employed decision trees and random forests to explore the salient psychosocial correlates of retirement expectations among Baby Boomers in two countries that differ in retirement norms and policies, US and South Korea. Sample included adults aged 52-65 from the 2016 wave of the Health and Retirement Study (n=1,192) and the Korean Longitudinal Study of Aging (n= 766). Expectation (probability) of working full-time for the next 5-10 years was modeled continuously (range: 0-100). Prediction models of expectations included 52 psychosocial factors relating to individual, family, and work characteristics. Mean expectations were higher in Koreans than US adults (71.4% vs. 36.7%). Age, self-reported probability of living to age 75, and work hours strongly predicted expectations in both US and Korean adults. While type of employment, Social Security or private pension benefits, and employment tenure were important predictors in US adults, flexible work arrangements were important in Koreans. Differences in features predicting retirement expectations between the US and Korean adults suggest opportunities for country-specific programs and policies aimed to promote workplace environment and pension benefits for successful retirement planning in the Baby Boom generation.

